# Atomistic simulation of carbohydrate-protein complex formation: Hevein-32 domain

**DOI:** 10.1038/s41598-019-53815-w

**Published:** 2019-12-12

**Authors:** Charles Oluremi Solanke, Dalibor Trapl, Zoran Šućur, Václav Mareška, Igor Tvaroška, Vojtěch Spiwok

**Affiliations:** 1 Department of Biochemistry and Microbiology, University of Chemistry and Technology, Prague, Technická 3, Prague 6, 166 28 Czech Republic; 2 Institute of Chemistry – Centre for Glycomics, Slovak Academy of Sciences, Dúbravská cesta 9, Bratislava, 845 38 Slovakia

**Keywords:** Molecular dynamics, Carbohydrates, Molecular modelling

## Abstract

Interactions between proteins and their small molecule ligands are of great importance for the process of drug design. Here we report an unbiased molecular dynamics simulation of systems containing hevein domain (HEV32) with N-acetylglucosamine mono-, di- or trisaccharide. Carbohydrate molecules were placed outside the binding site. Three of six simulations (6 × 2 μs) led to binding of a carbohydrate ligand into the binding mode in agreement with the experimentally determined structure. Unbinding was observed in one simulation (monosaccharide). There were no remarkable intermediates of binding for mono and disaccharide. Trisaccharide binding was initiated by formation of carbohydrate-aromatic CH/π interactions. Our results indicate that binding of ligands followed the model of conformational selection because the conformation of the protein ready for ligand binding was observed before the binding. This study extends the concept of docking by dynamics on carbohydrate-protein interactions.

## Introduction

Carbohydrate-protein interactions play an important role in many biological processes including immune response, inflammation, pathogen recognition, cell-cell interactions, cancer metastasis and many others^1^. Understanding the nature of these interactions is necessary for the design of new molecules for therapeutic intervention of these processes. However, carbohydrate-protein interactions are unique in many aspects, which makes them challenging to study by methods of molecular modeling and computational chemistry^2^.

First, carbohydrate-protein interactions are relatively weak compared to general drug-target interactions. Nature as well as scientists use the effect of multivalency to compensate this fact^3^. For example, multiple carbohydrate and protein molecules located on surfaces of cells interact much stronger than individual molecules. These interactions can also be influenced more efficiently by multivalent carbohydrate molecules, such as carbohydrate-modified dendrimers, nanoparticles, surfaces and glyco-clusters.

Second, carbohydrates are somewhat polar molecules. Polar interactions are in general more complicated to model than non-polar because they are more directional and because they are driven by a subtle interplay between solvation and desolvation.

Third, carbohydrates are quasi-symmetric with multiple OH groups in similar positions. As a result, several binding poses may have similar energy.

The most complete picture of the process of binding of a ligand on a protein is obtained by following it in real time. This is, at least in principle, possible by molecular dynamics simulation. Unfortunately, only a small fraction of protein-ligand complexes are formed in nanosecond to microsecond time scales, which are routinely accessible today^4^. Most protein-ligand complexes are formed in longer time scales and therefore cannot be studied by routine molecular simulations. Apart from its time scale limitations, the concept of docking by dynamics (or dynamic docking) has a great potential to accelerate the drug discovery process^4^.

Simulation of ligand binding can help us understand whether it is a one-step or rather a sequential process^4,5^. For a sequential process it is possible to determine all intermediate states. It is also possible to check how the binding of the ligand influences the structure of the protein (and *vice versa*) and what is the mechanism behind it (induced fit or conformational selection). Finally, atomistic simulation of protein-ligand binding can provide information about binding kinetics. All this information makes it possible to design ligands not only in terms of binding thermodynamics but also binding kinetics. Complementary, it is possible to explain mutations in a protein that affect binding, not only in terms of thermodynamics, but also in terms of kinetics.

As far as our knowledge goes, non-accelerated simulations of spontaneous formation of a carbohydrate-protein complex are very rare nowadays. An example of formation of a carbohydrate(-like molecule)-protein complex is the simulation of binding of inhibitors (Zanamivir, Oseltamivir) onto influenza neuraminidase^6^. However, these simulations were not pure unbiased simulations, because they were accelerated by multiscale approach combining molecular dynamics and Brownian dynamics simulation.

Here we present atomistic simulations of a carbohydrate-binding mini-protein (hevein domain HEV32^7^) in the presence of different oligosaccharides with examples of spontaneous formations of intermolecular complexes. The results were analysed to address general questions of protein-ligand interactions, such as the pathway of the process or mutual influence of binding molecules.

Hevein is a protein from latex tree (*Hevea brasiliensis*) involved in latex coagulation^8^. The molecule with 187 amino acids contains a carbohydrate-binding domain (also referred to as carbohydrate-binding module 18). It binds N-acetylglucosamine oligosaccharides on a latex glycoprotein present in latex particles^8^. It is also important latex allergen^8^.

This protein has been intensively studied as a model carbohydrate-binding protein. It was found that a 32-amino acid part is essential for carbohydrate binding^7^. This truncated protein is known as hevein-32 (HEV32). For its small size it ideal object for NMR^7,9^ peptide synthesis with unnatural amino acids^9^ and molecular simulations^9–11^. It binds N-acetylglucosamine trisaccharide ((GlcNAc)_3_) with a dissociation constant equal to approximately 10^−4^ (100 μM)^7^. Here we demonstrate that it is also an ideal model for molecular simulations of the formation of carbohydrate-protein complexes.

## Results

**Figure 1:**
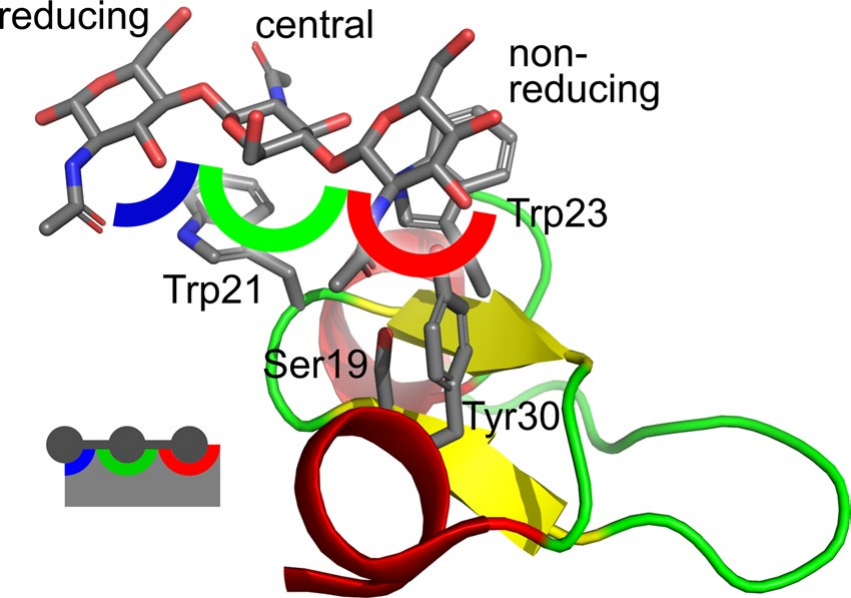
Binding sites in HEV32. Complex of HEV32 with (GlcNAc)_3_ (the first model of PDB ID 1T0W)^7^ with highlighted binding sites.

In the protein there are three subsites that bind individual monosaccharide moieties (Fig. 1). The first binding site (further depicted in red color) accommodates the non-reducing terminus. It is formed by residue Trp23 (via stacking CH/π interactions) together with two hydrogen-bonding residues (Tyr30 binding to 3-OH of the saccharide by its hydroxyl group and Ser19 binding to the carbonyl group of the acetyl moiety, also by its hydroxyl group). Tyr30 may also bind the methyl group of the acetyl moiety by CH/π interactions. The residue Trp21 forms the second binding site (further depicted in green color) (via stacking CH/π interactions) together with Glu1, which interacts with NH bond of acetyl and simultaneously with 3-OH group. This binding site is accommodated by the central residue of the trisaccharide. The last site (further depicted in blue color) is not defined by any clear noncovalent interaction except for a partial stacking with Trp21.

Fully atomistic unbiased simulations of systems containing the hevein domain HEV32, monosaccharide (β-d-GlcNAc*p*-OH, (GlcNAc)_1_), disaccharide (β-d-GlcNAc*p*-(1→4)-β-d-GlcNAc*p*-OH, (GlcNAc)_2_) or trisaccharide (β-d-GlcNAc*p*-(1→4)-β-d-GlcNAc*p*-(1→4)-β-d-GlcNAc*p*-OH, (GlcNAc)_3_) and 2271–2287 water molecules were carried out. A saccharide was placed manually to space outside the binding site using UCSF Chimera^12^. Each production simulation (2 μs) was done in duplicates differing in the mutual carbohydrate and protein orientation at the beginning of the simulation. Trajectories (without water) of all simulations are available online via Materials Cloud (10.24435/materialscloud:2019.0042/v2).

**Figure 2:**
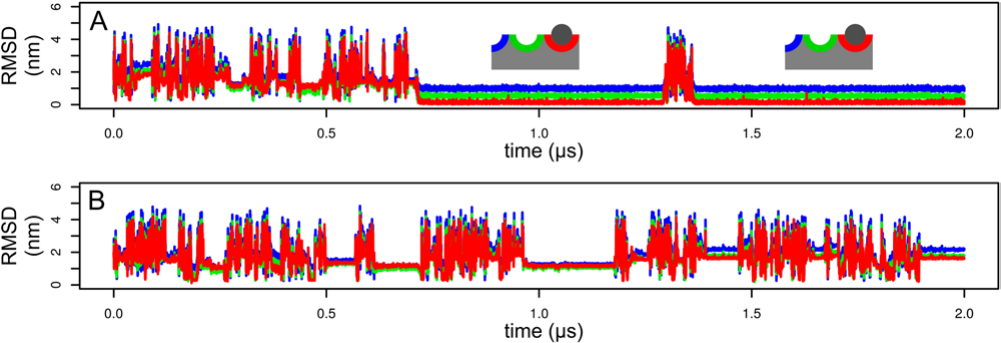
Simulation of (GlcNAc)_1_ binding. RMSD profile of the monosaccharide moiety in the first (**A**) and the second (**B**) replica of the simulation. The first replica resulted in a specific binding depicted schematically. The second replica did not led to a specific binding.

The results of the simulations were monitored by RMSD profiles. Briefly, protein atoms of trajectory snapshots were fitted onto NMR structure^7^, and positions of monosaccharide moieties were compared with individual monosaccharide moieties in the NMR structure. Details of RMSD calculations can be found in Methods. These profiles are depicted in Fig. 2 for simulations of the complex with (GlcNAc)_1_. The profile clearly shows short periods during which RMSD exceeds 4 nm. These periods represent the entirely unbound state. Periods of high RMSD are separated by similarly long periods during which RMSD drops to 1–2 nm and is relatively stable. These periods represent nonspecific binding. Specific binding was observed at the time 720 ns in the first replica of the simulation (Fig. 2A). The molecule of (GlcNAc)_1_ docked into the “red” binding site. RMSD values were lowest for the “red” binding site (red trace), higher for the “green” and highest for the “blue” binding site. This complex dissociated at the time approximately 1290 ns and docked again at the time approximately 1360 ns. Visual inspection of binding of (GlcNAc)_1_ revealed that the process is rather one-step with no distinct intermediates. The first binding is initiated by an interaction of the methyl group of the acetyl moiety of (GlcNAc)_1_ with Tyr30, followed by a rotation of the ligand into the correct binding mode. The second binding was initiated by the formation of CH/π interactions between the monosaccharide and Trp23.

The results for the second replica of the simulation (Fig. 2B) were similar to the results of the first one, except that no specific binding was observed.

**Figure 3:**
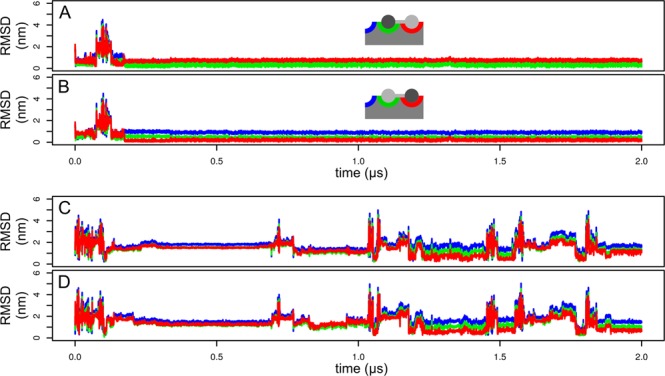
Simulation of (GlcNAc)_2_ binding. RMSD profile of the monosaccharide moieties in the first (**A**,**B**) and the second (**C**,**D**) replica of the simulation calculated for the reducing (**A**,**C**) and the nonreducing (**B**,**D**) monosaccharide moiety. The first replica resulted in a specific binding depicted schematically. The second replica did not led to a specific binding.

RMSD profiles for simulations of the complex with (GlcNAc)_2_ are depicted in Fig. 3. In the first replica of the simulation (Fig. 3A,B) there were two short periods of nonspecific binding separated by a short period of complete unbinding. Visual inspection of the nonspecific binding period revealed that (GlcNAc)_2_ bound to the indole moieties of tryptophans via CH/π interactions, but via the opposite face of monosaccharide moieties. In this nonspecific binding mode the nonreducing terminus interacts by CH bonds on atoms C1, C3 and C5 and the reducing terminus interacts by CH bonds on atoms C2, C4 and C6. In the specific binding mode it is opposite (the nonreducing terminus interacts by CH bonds on atoms C2, C4 and C6 and the reducing terminus interacts by CH bonds on atoms C1, C3 and C5).

At the end of the second nonspecific binding period (at time 175 ns) the molecule of (GlcNAc)_2_ bound specifically. Visual inspection of the trajectory revealed that the binding takes place by the relatively fast rotation of the ligand. The nonreducing residue docked into the “red” binding site (red trace in Fig. 3B). The reducing residue docked into the “green” binding site (green trace in Fig. 3A). The complex was stable for the rest of the simulation.

**Figure 4:**
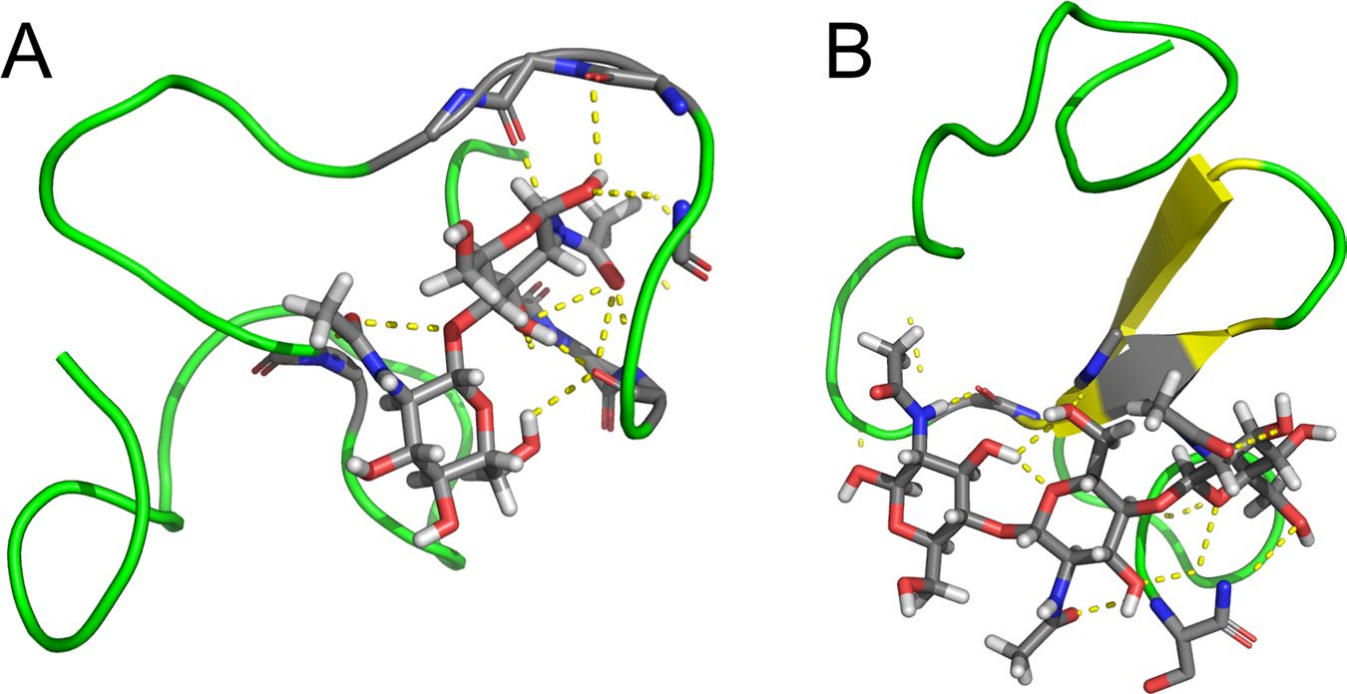
Nonspecific binding. Snapshot of the simulation of the complex with (GlcNAc)_2_, the second replica at 500 ns (**A**) and the complex with (GlcNAc)_3_, the first replica at 1000 ns (**B**).

Specific binding was not observed in the second replica of the simulation (Fig. 3C,D). There was a long period (approximately 500 ns) of nonspecific binding characterized by binding of the ligand onto the opposite side of the protein (Fig. 4A). The molecule of (GlcNAc)_2_ forms numerous hydrogen bonds mostly with backbone atoms. The residue Tyr30 distracted from its original position. The similar transition was observed in previous simulations the HEV32^11^.

**Figure 5:**
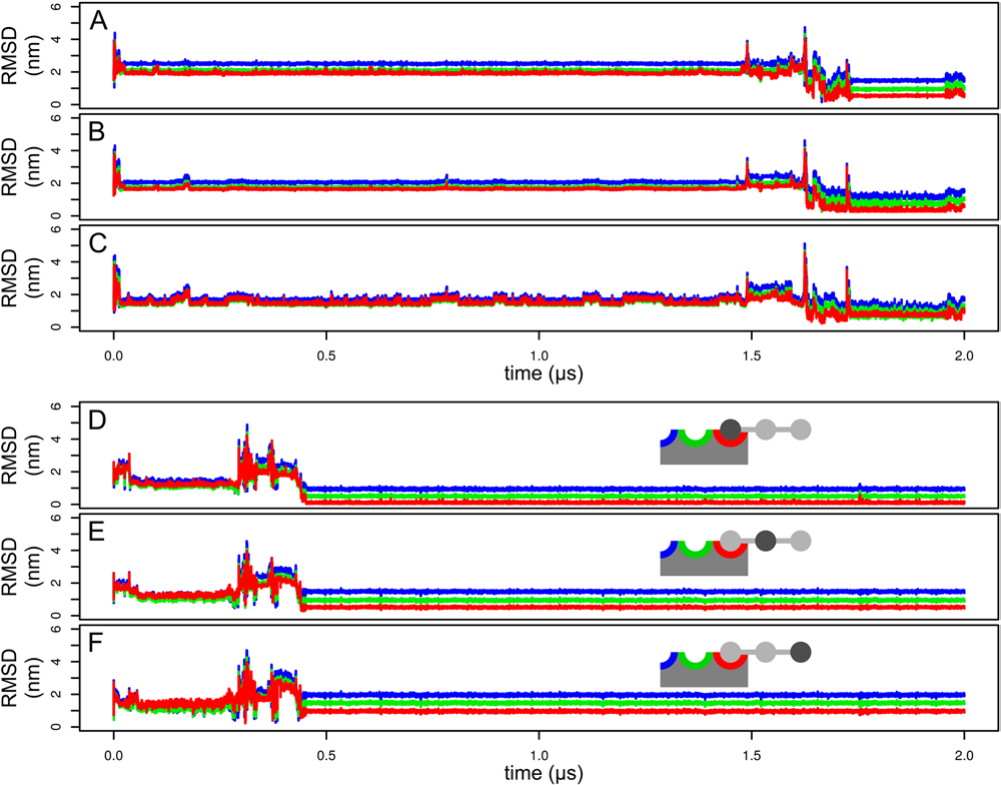
Simulation of (GlcNAc)_3_ binding. RMSD profile of the monosaccharide moieties in the first (**A**–**C**) and the second (**D**–**F**) replica of the simulation calculated for the reducing (**A**,**D**), internal (**B**,**E**) and nonreducing (**C**,**F**) monosaccharide moiety. The first replica resulted to a non-specific binding. The second replica led to a specific binding depicted schematically.

In the first replica of the simulation with (GlcNAc)_3_ (Fig. 5A–C) we observed relatively fast (at the time 25 ns) nonspecific binding at the opposite side of the protein (Fig. 4B). This nonspecific binding mode dissociated at approximately 1500 ns. It was characterized by multiple hydrogen bonds between the ligand and the backbone of the protein, similarly to the binding mode observed in the second replica of the simulation of the complex with (GlcNAc)_2_.

In the second replica of the simulation with (GlcNAc)_3_ (Fig. 5D–F) we observed a nonspecific binding after approximately 70 ns. The ligand was bound via nonspecific hydrogen bonds and CH/π interactions with Tyr30 and Trp21. This assembly dissociated at the time about 290 ns and at the time 444 ns the ligand bound specifically. This complex was stable for the rest of the simulation. It was characterized by binding of the reducing terminus of (GlcNAc)_3_ in the “red” subsite. The central monosaccharide moiety was interacting with backbone atoms of the protein and with Gln2. There were only a few contacts between the protein and the non-reducing terminus.

The binding process in the second replica was initiated by CH/π interactions of the central monosaccharide moiety with Trp23. The orientation of the central moiety was not suitable for specific binding because CH/π interactions were formed by atoms C1, C3 and C5, whereas interaction via C2, C4 and C6 is required for binding into the “red” subsite. After this initial binding, the ligand slid on the surface of the indole ring of Trp23 and the reducing terminus docked into the “red” subsite. Since the reducing monosaccharide moiety is oriented in an opposite way relative to the central moiety, the reducing moiety formed CH/π interactions via its C2, C4 and C6 atoms.

**Figure 6:**
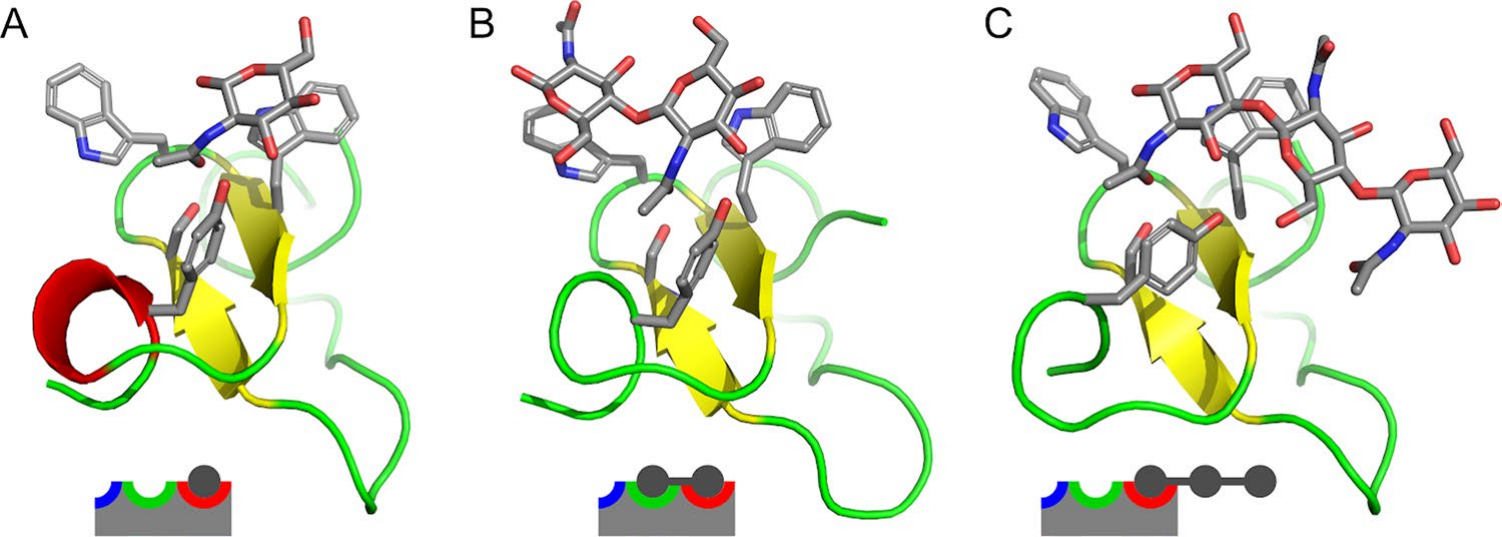
Structures of bound states with schematic views. (**A**) Time 1000 ns of the first replica of the simulation with (GlcNAc)_1_; (**B**) time 1000 ns of the second replica of the simulation with (GlcNAc)_2_; (**C**) time 1000 of the second replica of the simulation with (GlcNAc)_3_.

Correct bound states are summarized in Fig. 6. This figure depicts the state at 1000 ns of simulations of (GlcNAc)_1_ (the first replica), (GlcNAc)_2_ (the second replica) and (GlcNAc)_3_ (the second replica). As expected, binding of a monosaccharide residue into the “red” subsite was observed in all three cases and is likely to be essential for binding.

**Figure 7:**
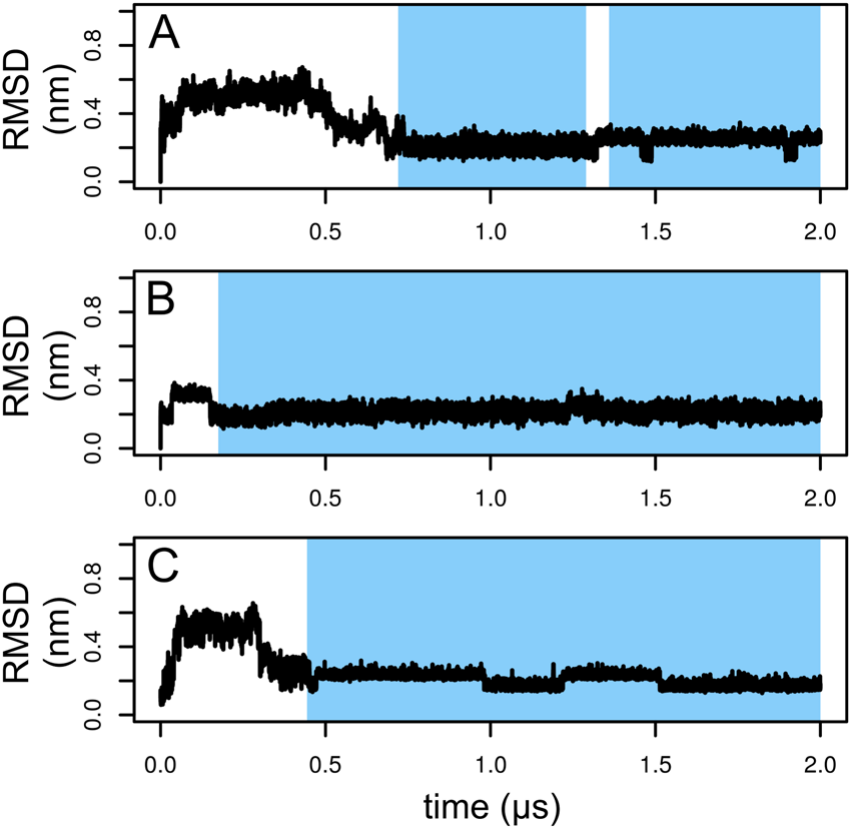
Evolution of RMSD of key binding residues (Ser19, Trp21, Trp23 and Tyr30) in the first replica of the simulation of the complex with (GlcNAc)_1_ (**A**), the first replica of the complex with (GlcNAc)_2_ (**B**) and the second replica of the complex with (GlcNAc)_3_ (**C**). Bound states are highlighted by blue color.

The question arises whether the structure of the protein is influenced by binding of a ligand and what is the mechanism of such influence. This was investigated by calculation of RMSD profiles for key binding residues (Ser19, Trp21, Trp23 and Tyr30) with the initial structure as a reference. Figure 7 shows RMSD profiles of simulations in which binding was observed. It clearly shows that the binding of the ligand stabilizes the binding site (low RMSD values around 0.2 nm). Interestingly, in all cases RMSD drops to approximately 0.2 nm before binding, i.e. it first adopts the structure suitable for binding and then the binding takes place.

## Discussion

The result of the simulation of protein-ligand binding can be influenced by the initial position of both binding partners. Placement of both molecules close to each other and in an orientation favorable for binding may positively influence the chance of observation of complex formation. This may bias kinetics in favor of binding. To test whether the initial position of both binding partners may influence binding kinetics we inspected RMSD profiles. They clearly show that in the period between the start of the simulation and ligand binding the value of RMSD changes rapidly. This clearly indicates that the protein and ligand explore a wide range of mutual orientations and the binding process is independent (aside from the deterministic nature of molecular simulations) on the initial ligand position. This is also clearly visible from visual inspection of trajectories.

Interestingly, the bound state of (GlcNAc)_3_ is characterized by the presence of the reducing terminus in the “red” subsite. This is likely to be a local minimum of binding because of few protein-ligand contacts. The binding pose with the reducing terminus in the “green” or “blue” subsite is likely to be the global minimum. This illustrates the limitation of two microsecond dynamics simulation in the determination of the bound state of an oligomeric ligand. Longer simulation or simulations in a higher number of replicas would be necessary to address this problem.

From the volume of the simulation box it is possible to calculate concentrations of protein and saccharidic ligand as approximately 24 mmol/l, which corresponds to concentration of protein about 80 mg/ml and of carbohydrate ((GlcNAc)_3_) approximately 30 mg/ml. This is significantly higher than the concentrations used in the experiment. However, protein and/or ligand solubility are not issues in biomolecular simulations due to a periodic boundary condition algorithm, which minimizes protein-protein or ligand-ligand interactions. According to the experimental value of dissociation constant of (GlcNAc)_3_ concentrations during the simulation correspond to 94% saturation of the complex.

As far as we are aware, dissociation constants for (GlcNAc)_1_ and (GlcNAc)_2_ are not available in the literature. We have observed one unbinding in the simulation of the complex with (GlcNAc)_1_, so we can roughly estimate the dissociation constant. (GlcNAc)_1_ stayed in the bound state for approximately 60% of the time (30% taking both replicas into an account). This (60 or 30% saturation) corresponds to *Kd* = 0.006 (6 mM) or 0.04 (40 mM), respectively. However, it must be kept in mind that this estimate was made based on two binding and one unbinding event and more binding and unbinding events would be required to make an accurate estimate.

Visual inspection of binding of all ligands did not reveal any common mechanism of binding. Binding of (GlcNAc)_1_ and (GlcNAc)_2_ was fast with barely remarkable intermediates. Binding of (GlcNAc)_3_ was characterized by the formation of CH/π interactions with a “wrong” monosaccharide moiety, followed by sliding of (GlcNAc)_3_ allowing binding of the “correct” monosaccharide moiety. This indicates that CH/π interactions^13,14^ play an essential role not only in stabilizing carbohydrate-protein assemblies, but also in the binding process and initial carbohydrate recognition.

Tyr30 is an interesting amino acid residue of HEV32. This residue acts as an acceptor of CH/π interactions with the acetyl moiety of GlcNAc. It also forms a classical H-bond via its OH group. It was involved in the initial recognition of (GlcNAc)_1_ and (GlcNAc)_2_. In the second replica of the simulation of the residue Tyr30 distracted from its original position. This was followed by a rearrangement of the C-terminal part of HEV32. A similar transition has been observed in a previous simulation of HEV32^11^. It is not clear whether this transition plays any biological role or is the result of artificial truncation of hevein into smaller HEV32.

The fastest binding of the ligand was observed for (GlcNAc)_2_, followed by (GlcNAc)_3_ and (GlcNAc)_1_ (the first binding). We can speculate that there are two contradicting effects. Binding of larger ligands such as (GlcNAc)_3_ is slowed down by their larger size, which makes the search of the binding site slow due to many opportunities for nonspecific binding. On the other hand, this binding is accelerated by a high affinity due to high number of intermolecular interactions. The situation is the opposite for small ligands such as (GlcNAc)_1_.

Figure 7 clearly shows that upon binding of a saccharide ligand, the structure of the binding site is stabilized. The question is whether this happens via the model of induced fit or conformational selection. The former model assumes that binding of the ligand actively causes a conformational change in the protein. The later model assumes that the protein exists in solution as a pool of different conformations and the ligand picks those in the conformation suitable for binding. The value of RMSD in simulations with binding events always reached the value of approximately 0.2 nm shortly before the ligand binding event. This supports the model of conformational selection. We can therefore speculate that the binding takes place via the conformational selection model.

In our experience, many successful unbiased simulations of the formation of a protein-ligand complex reported in literature represented simulations of the binding of charged ligands^4^. It is possible that the initial phase of binding characterized by random search of the entrance to the binding site by the ligand is more accessible when the ligand and the protein binding sites carry opposite charges. This can be explained by a long reach of electrostatic interactions. More simulations of protein-ligand binding would be necessary to verify this trend. Interestingly, ligands simulated in this study were neutral.

The question is whether it is possible to use an atomistic simulation as a tool to locate a carbohydrate-binding site. Some of the simulations in this study resulted in long and stable, but nonspecific binding poses. These nonspecific binding poses would be difficult to distinguish from the specific binding site in the situation when the specific binding site is not known. Especially, RMSD profiles from the first replica of the simulation of the complex with (GlcNAc)_3_ are difficult to distinguish in terms of stability from a specific binding. However, there are two characteristics that may indicate nonspecific binding. First, saccharides interacted almost only with backbone atoms (N–H and C=O groups) of the protein in nonspecific binding modes. There were no such interactions in the specific binding mode of HEV32. Second, CH/π interactions are characteristic for carbohydrate-protein interactions, but they were absent in nonspecific binding modes observed in this study. Presence of CH/π interactions can be used to support the identification of a specific binding mode. More studies would be necessary to verify these signatures of specific carbohydrate-protein complexes.

In conclusion, atomistic simulations with the design presented in this study have a great potential to elucidate the mechanism of carbohydrate-protein interactions. Development of fast computers and enhanced sampling methods is likely to extend the size of proteins from HEV32 mini-protein to larger biomolecules.

## Methods

All simulations were carried out using GROMACS 5.1.3^15^ with GPU acceleration. The NMR structure of a truncated hevein domain (hevein-32, PDB ID: 1T0W^7^, model 1) was used as the starting structure. Protein was simulated using Amber99SB-ILDN force field^16^. Carbohydrates ((β-d-GlcNAc)_n_-OH, n = 1, 2, 3, referred to as (GlcNAc)_1_, (GlcNAc)_2_ and (GlcNAc)_3_, respectively) were modeled using Glycam 06j force field^17^. The program *acpype*.*py*^18^ was used to convert Glycam topology to Gromacs formats. Water was modeled using the TIP3P model. Time step was set to 2 fs and all bonds were constrained by the LINCS algorithm^19^. Electrostatics was modeled using the particle-mesh Ewald (PME) method with cut-off set to 1 nm^20^. The temperature was kept constant (300 K) by Parrinello-Bussi thermostat^21^. Each system was optimized by energy minimization. Next, each system was equilibrated by 300 ps simulation in the NPT ensemble (constant number of particles, pressure and temperature). All non-hydrogen atoms of the protein and a ligand were restrained by a harmonic restraint (*k* = 1000 kJ mol^−1^ nm^−2^) to their original positions during this simulation. Finally, 2 μs production simulation in the NVT ensemble (constant number of particles, volume and temperature) was performed for each system.

Analysis of trajectories was done by monitoring a root-mean-square deviation (RMSD) from the reference NMR structure (after energy minimization). NMR structure (PDB ID: 1T0W^7^, model 1) contains a (GlcNAc)_3_ bound in the binding site. Seven atoms of each monosaccharide (C1 to C6 together with O5) were extracted from the NMR structure and used as a reference together with the protein structure. RMSD was calculated by fitting the trajectory onto non-hydrogen atoms of the protein, followed by the calculation of RMSD on the seven selected atoms of a respective monosaccharide moiety.
